# Association of total lifetime breastfeeding duration with midlife handgrip strength: findings from Project Viva

**DOI:** 10.1186/s12905-022-01880-1

**Published:** 2022-07-23

**Authors:** Irasema C. Paster, Pi-i D. Lin, Sheryl L. Rifas-Shiman, Wei Perng, Jorge E. Chavarro, Emily Oken

**Affiliations:** 1 A.T. Still University School of Osteopathic Medicine in Arizona, Mesa, AZ USA; 2 Division of Chronic Disease Research Across the Lifecourse, Department of Population Medicine, Harvard Medical School and Harvard Pilgrim Health Care Institute, 401 Park Drive, Suite 401, Boston, MA 02215 USA; 3 Department of Nutrition, Harvard T.H. Chan School of Public Health, Boston, USA; 4 Department of Epidemiology, Lifecourse Epidemiology of Adiposity and Diabetes (LEAD) Center, Colorado School of Public Health, University of Colorado Anschutz Medical Campus, Aurora, CO USA

**Keywords:** Breastfeeding, Hand grip strength, Lactation, Muscle mass

## Abstract

**Background:**

Lactation has long term effects on maternal health, but the relationship between lactation and long-term handgrip strength, a marker of musculoskeletal function and healthy aging, has not been explored.

**Objective:**

Examine the relationship between total lifetime breastfeeding duration (BFD) and midlife handgrip strength.

**Methods:**

We measured handgrip strength as a marker of overall strength among 631 women in the Project Viva cohort. At the same visit, women reported their BFD for each birth, and we derived total lifetime BFD. We used multivariable linear regression models to estimate associations of lifetime BFD in months with midlife handgrip strength in kilograms, adjusted for race/ethnicity, education, marital status, household income, age at first pregnancy and age at handgrip strength assessment.

**Results:**

Mean (standard deviation) age was 50.7 (5.1) years, lifetime BFD was 21.6 (19.5) months, and handgrip strength was 28.0 kg (6.0) in the dominant and 26.0 kg (5.6) in the non-dominant hand. In fully adjusted models, each 3-month increment in lifetime BFD was associated with 0.10 kg (95% CI 0.02, 0.18) higher handgrip strength for the dominant hand and 0.10 kg (95% CI 0.03, 0.18) for the nondominant hand. Results were similar in models examining mean BFD per pregnancy rather than total BFD. There was no evidence of effect modification by race/ethnicity.

**Conclusions:**

Our study suggests that there is a small beneficial effect of lifetime BFD on handgrip strength. Future studies can explore mechanisms by which BFD affects body composition and associations with other outcomes related to lean mass such as sarcopenia.

**Supplementary Information:**

The online version contains supplementary material available at 10.1186/s12905-022-01880-1.

## Background

In addition to documented benefits for the child [1], breastfeeding has many benefits for the mother including associations with lower future risks of various types of cancer, type 2 diabetes mellitus, hyperlipidemia and rheumatoid arthritis [2]. It has also been hypothesized that lactation may play a role in muscle mass retention as women age through a rise in estrogen during the lactation period [3, 4]. Additionally, while bone mineral density decreases during lactation, this decrease is transient and most women recover bone density after lactation has ended with no significant long-term skeletal changes [5]. These benefits for body composition may yield metabolic benefits. For example, in actively breastfeeding women, longer breastfeeding duration is associated with improved metabolic markers [2].

Handgrip strength is an inexpensive and validated method of analyzing muscle strength and mobility, particularly in older populations [6]. Stronger handgrip is predictive of higher cognition, physical functioning, and mobility, as well as lower mortality in older populations [6]. In women, lower handgrip strength has been associated with an increased risk of osteosarcopenia [7] and is one of the major criteria for diagnosis of sarcopenia [8], or age-related loss of muscle mass [9]. Less is known about potentially modifiable determinants of handgrip strength such as breastfeeding, despite the growing recognition of its clinical importance. One study analyzed data from the Korea National Health and Nutrition Examination Survey 2010–2011 and found that the odds ratios of developing sarcopenia decreased with increasing breastfeeding duration [10]. To our knowledge, that was the only study that has examined this relationship and there is a need to explore this topic further.

The objectives of this study were to examine the extent to which total lifetime breastfeeding duration was associated with midlife handgrip strength. We hypothesized that total lifetime breastfeeding duration would have a positive association with midlife handgrip strength. Our second objective was to examine whether the association of lifetime breastfeeding duration with midlife handgrip strength varied by race/ethnicity in light of documented differences in breastfeeding duration across racial/ethnic groups [11–13]. We hypothesized that, although both breastfeeding duration [12, 13] and body composition [14] have been shown to vary by race/ethnicity, there would not be a difference in association between lifetime breastfeeding duration and midlife handgrip strength among women in different racial/ethnic groups.

## Methods

### Study population

We used data from women who participated in Project Viva for this analysis. Project Viva is a prospective cohort study that recruited pregnant women carrying a singleton pregnancy during their initial obstetric care visit at Atrius Harvard Vanguard Medical Associates in eastern Massachusetts between 1999 and 2002 [15]. Study exclusion criteria included multiple gestation, inability to answer questions in English, gestational age ≥ 22 weeks at recruitment, and plans to move away from the study area before delivery [15]. There were 2128 births to 2100 mothers (28 women enrolled with more than one pregnancy). Project Viva attempted to follow-up all participants thereafter, most recently at a mid-life follow-up study visit at a mean (SD) of 18.2 (0.6) years after study enrollment, conducted 2017–2021. [15]

For this analysis, we included Project Viva women who reported information on lifetime breastfeeding duration and were tested for handgrip strength at the midlife visit. A Project Viva research assistant administered pre-test questions. If the participant 1) had hand or wrist surgery on both hands within the last three months or 2) was unable to hold the dynamometer with both hands (e.g., missing arms, hands, or thumbs on both hands; paralysis of both hands) then the research assistant did not measure handgrip strength. If the participant had hand or wrist surgery on one hand within the last 3 months or was unable to hold the dynamometer with one hand, they performed the handgrip strength exam only using the opposite hand. Of the 2100 women enrolled in Project Viva, 676 provided handgrip strength data at the midlife visit and 631 provided lifetime breastfeeding duration data. The participants included in this analysis had similar socioeconomic statuses and race/ethnicity proportions as those without mid-life follow-up data, however there was a slightly larger percentage of college graduates in those included versus excluded (75% vs. 60%) (Additional file 2: Table S1).

### Exposure

We defined the primary exposure variable as total lifetime breastfeeding duration across all pregnancies, measured continuously per 3-month increment. At the midlife visit, women reported information about all lifetime pregnancies (not limited to the Project Viva index pregnancy) via questionnaire, including year of pregnancy (from which we calculated age at first pregnancy) and duration of breastfeeding for each pregnancy that resulted in a live birth. We calculated total lifetime breastfeeding duration by adding the total duration for each reported pregnancy. We also calculated total lifetime breastfeeding duration ranked into quartiles (with quartile 1 as the reference category), dichotomous ever breastfed (yes vs. no) and average breastfeeding duration per live birth (per 3-month increment).

### Outcome

The outcome variable was midlife handgrip strength. Trained research assistants measured handgrip strength at the midlife visit in kilograms using a Jamar dynamometer. The Jamar dynamometer is a validated instrument for measuring handgrip strength and serves as a reference standard across clinical and epidemiological studies [16, 17]. Using the American Society of Hand Therapists protocol, participants were instructed to squeeze the dynamometer with maximal effort for three separate attempts per hand with thirty seconds of rest in between to avoid muscle fatigue [16]. We used the average measurement of the three attempts separately for the dominant and non-dominant hand in our analyses and also calculated the average of all 6 measures.

### Covariates

We considered a priori covariates that may be associated with both the exposure and outcome in our analysis, based on review of the literature and using directed acyclic graphs. Women reported annual household income via a self-administered questionnaire [15]. At study enrollment, women reported, highest educational attainment, and marital status via interview. At study enrollment, research assistants also asked mothers: “Which of the following best describes your race or ethnicity?” Mothers had a choice of one or more of the following mutually exclusive racial/ethnic groups: Hispanic or Latina, White or Caucasian, Black or African American, Asian or Pacific Islander, American Indian or Alaskan Native, and other (please specify). For the participants who chose “other” race/ethnicity, we compared the specified responses to the U.S. census definition for the other five races and ethnicities and reclassified them where appropriate. If a participant chose more than one racial/ethnic group, we coded them as “more than 1 race/ethnicity”. We chose to adjust for race/ethnicity because we consider it to be a social construct that may affect breastfeeding duration through a variety of mechanisms; there are also differences in body composition proportions across race/ethnicity [14]. We also chose to adjust for age at first pregnancy because it is inversely related to lifetime breastfeeding duration and could potentially confound the association between lifetime breastfeeding duration and midlife handgrip strength [18]. At enrollment, pregnant women reported their diet during the index pregnancy using a validated food frequency questionnaire [19, 20]. The food frequency questionnaire used in Project Viva was modified for pregnant adults from the Willett FFQ used in the Nurses’ Health Study and other large cohort studies [15, 21, 22]. For our analyses, we calculated Alternate Healthy Eating Index, slightly modified for pregnancy (AHEI-P) [35] as a measure of overall diet quality. At enrollment women also reported pre-pregnancy physical activity using a questionnaire modified from the Physical Activity Scale for the Elderly (PASE) [23]. Women were asked to recall their weekly activity over the year before pregnancy and to report average hours per week of activity [23]. Total physical activity was treated as a continuous covariate (hours/week). At the midlife visit, women reported whether they had ever smoked cigarettes. Older age is inversely associated with handgrip strength measurements [24]. Therefore, age at handgrip strength assessment was identified as a precision covariate for handgrip strength because it accounts for variation in handgrip strength.

We considered pre-pregnancy weight as a covariate as previous studies have found that BMI has a positive association with handgrip strength [25, 26]. We could not adjust for weight prior to all pregnancies, as we did not collect this variable. We did collect estimated weight status at 10 years of age, reported at study enrollment, but after adjusting for it in a sensitivity analysis, it did not meaningfully change the results, so we did not include this variable in our models.

Lifetime parity was found to be moderately associated with increased lifetime breastfeeding duration (Spearman r = 0.33, *p* < 0.0001). We accounted for parity by using average breastfeeding duration per live birth.

### Analysis

In our primary analysis, we estimated associations of lifetime breastfeeding duration in months (continuous, reported per 3-month increment to make estimates meaningful in magnitude) with midlife handgrip strength in kilograms using unadjusted (Model 1) and multivariable adjusted linear regression. We tested for normality of the exposure distribution and decided to use the non-transformed lifetime breastfeeding duration for ease of interpretation as both the log_2_ transformed and nontransformed lifetime breastfeeding duration yielded models with normally distributed residuals. We adjusted for race/ethnicity, education, marital status, ever smoking, household income at enrollment, and mother’s age at first pregnancy (Model 2). Model 3 was also adjusted for age at handgrip strength measurement due to the inverse relationship between age and handgrip strength [24]. Finally, in sensitivity analyses, we additionally adjusted for diet (AHEI-P [19], units) and pre-pregnancy physical activity (hours/week) as both can impact maternal body composition and therefore impact breastfeeding outcomes [26–29]. However, because we had information on pre-pregnancy diet and physical activity only for the index pregnancy, we restricted this sensitivity analysis to women who enrolled in Project Viva at their first pregnancy (Model 4). We then repeated all regression models using quartiles of lifetime breastfeeding duration in months as the exposure and compared differences in mean handgrip strength using the first quartile as the reference.

To assess for racial/ethnic differences in exposure-outcome associations, we added an interaction term between lifetime breastfeeding duration and race/ethnicity and also examined results in models stratified by race/ethnicity. We considered evidence for significant interaction if the interaction *p* value was < 0.15.

We repeated Models 1 to 4 using the exposure average breastfeeding duration per live birth as a standardized indicator of breastfeeding duration irrespective of parity.

We performed all analyses using SAS version 9.4 (Cary, NC). Due to the small number of missing covariate values, we did not use multiple imputation and allowed the sample size to decrease slightly across multivariable models.

## Results

Selected participant characteristics are presented in Table 1. The mean (SD) age of women in the sample at the time of handgrip strength measurement was 50.7 years (SD: 5.1). The study population included 14% who identified as Black, 7% Hispanic/Latina, 6% Asian, 68% White, and 5% other race/ethnicity.

**Table 1:** Characteristics of Project Viva women included in this analysis according to lifetime breastfeeding status

	Overall	Never breastfed	Ever breastfed	*P* value
	N (%) or mean (SD)			
	n = 631	63 (10)	568 (90)	
Race/ethnicity, %				0.489
Black	90 (14)	8 (13)	82 (14)	
Hispanic	44 (7)	4 (6)	40 (7)	
Asian	39 (6)	2 (3)	37 (7)	
White	426 (68)	48 (76)	378 (67)	
Other	30 (5)	1 (2)	29 (5)	
College graduate, %				0.004
No	155 (25)	25 (40)	130 (23)	
Yes	474 (75)	38 (60)	436 (77)	
Household income > $70,000/year, %				0.115
No	197 (34)	26 (43)	171 (33)	
Yes	379 (66)	34 (57)	345 (67)	
Age at 1st pregnancy, years	28.5 (6.1)	28.5 (6.8)	28.5 (6.0)	0.745
Nulliparous at index pregnancy, %				0.133
No	327 (52)	27 (43)	300 (53)	
Yes	304 (48)	36 (57)	268 (47)	
Married or cohabiting, %				0.096
No	47 (7)	8 (13)	39 (7)	
Yes	582 (93)	55 (87)	527 (93)	
Dominant hand				0.649
Right	570 (90)	55 (87)	515 (91)	
Left	51 (8)	7 (11)	44 (8)	
Handgrip strength measurements, kg				
Dominant hand	28.0 (6.0)	28.2 (6.6)	28.0 (6.0)	0.834
Non-dominant hand	26.0 (5.6)	26.0 (6.1)	26.0 (5.6)	0.999
Average of dominant and non-dominant hands	27.0 (5.6)	27.1 (6.1)	27.0 (5.6)	0.898
Age at handgrip strength measurement, years	50.7 (5.1)	50.0 (5.9)	50.8 (5.0)	0.266
Parity at handgrip strength measurement, births	2.5 (1.0)	2.2 (0.9)	2.5 (1.0)	0.951
Ever smoking, %				0.075
No	474 (78)	42 (69)	432 (79)	
Yes	135 (22)	19 (31)	116 (21)	

At study enrollment in the index pregnancy, 75% of women reported having a college degree and 93% were married or cohabitating. Their mean lifetime breastfeeding duration, reported at the midlife visit, was 21.6 months (SD: 19.5) and the majority (90%) of the women reported breastfeeding following at least one pregnancy. Mean midlife handgrip strength was 28.0 kg (SD: 6.0) for dominant hand and 26.0 kg (SD: 5.6) for nondominant hand, which were comparable to the normative reference values for women in the United States. [30]

**Fig. 1:**
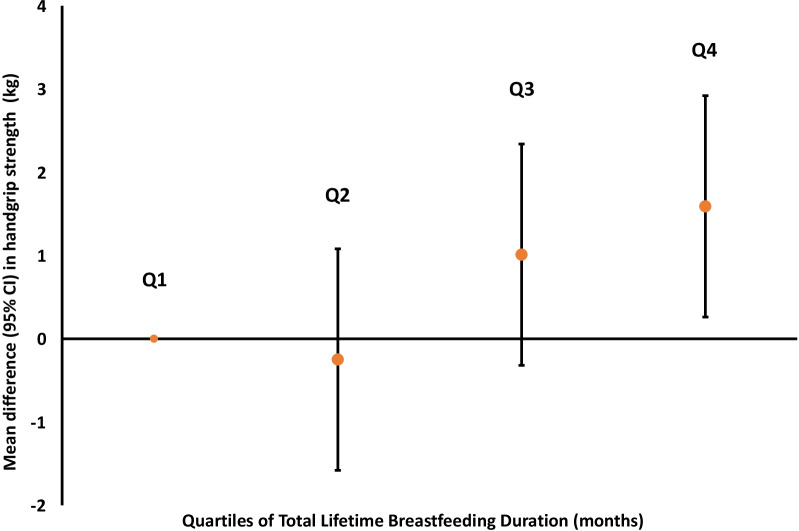
Adjusted* associations of total lifetime breastfeeding duration (quartiles) with average of dominant and nondominant handgrip strength (kg). *Adjusted for race/ethnicity, education, marital status, ever smoker, household income at enrollment, age at 1st pregnancy, and age at handgrip strength (Model 3). Quartile (Q) of total lifetime breastfeeding duration [median, interquartile range (IQR) and range (min–max)]: Q1: 2 months (IQR: 0–5 months, range: 0–7 months), Q2: 12 months (IQR: 9.8–13 months, range 8–17 months), Q3: 24 months (IQR: 20–26 months, range 18–30 months), Q4: 42 months (IQR: 36–57 months, range 31–100 months), confidence interval (CI)

The results of the linear regression models are shown in Table 2 and Fig. 1. Overall, in the fully adjusted models we found that each 3-month increment in lifetime breastfeeding duration was associated with 0.10 kg (95% CI 0.02, 0.18) higher handgrip strength for the dominant hand and 0.10 kg (95% CI 0.03, 0.18) for the nondominant hand, adjusting for race/ethnicity, marital status, ever smoking, household income at enrollment, age at first pregnancy, and age at the handgrip strength measurement (Model 3). Using quartiles as the exposure, we observed higher handgrip strength comparing the 4th quartile of breastfeeding duration to the first quartile (Table 2), but the estimates were statistically significant only for the non-dominant hand. Additional adjustments for pre-pregnancy physical activity and maternal diet did not substantially change the effect estimates among N = 304 women who were nulliparous at enrollment (Table 2, Model 4 and Additional file 3: Table S2).

**Table 2:** Associations of lifetime breastfeeding duration (BFD) with dominant and nondominant handgrip strength, kg

	Model 1 β (95% CI)	Model 2 β (95% CI)	Model 3 β (95% CI)	Model 4 β (95% CI)
*Dominant hand*				
Exposure				
Lifetime BFD, per 3 months	**0.09 (0.01, 0.16)**	**0.09 (0.02, 0.17)**	**0.10 (0.02, 0.18)**	**0.15 (0.03, 0.28)**
Lifetime BFD, Quartiles				
Q1	0.0 (ref)	0.0 (ref)	0.0 (ref)	0.0 (ref)
Q2	0.23 (− 1.10, 1.57)	− 0.34 (− 1.78, 1.09)	− 0.33 (− 1.76, 1.10)	0.17 (− 1.86, 2.19)
Q3	1.28 (− 0.05, 2.61)	0.98 (− 0.45, 2.41)	0.96 (− 0.47, 2.38)	1.18 (− 0.99, 3.35)
Q4	1.31 (− 0.03, 2.64)	1.34 (− 0.10, 2.78)	1.41 (− 0.03, 2.84)	**2.33 (0.10, 4.56)**
Trend-p*	0.021	0.021	0.017	0.027
Average BFD per live birth, per 3 months	0.08 (− 0.09, 0.25)	0.13 (− 0.06, 0.31)	0.15 (− 0.03, 0.34)	0.16 (− 0.12, 0.43)
*Nondominant hand*				
Exposure				
Lifetime BFD, per 3 months	**0.09 (0.02, 0.16)**	**0.10 (0.03, 0.17)**	**0.10 (0.03, 0.18)**	**0.15 (0.03, 0.26)**
Lifetime BFD, Quartiles				
Q1	0.0 (ref)	0.0 (ref)	0.0 (ref)	0.0 (ref)
Q2	0.47 (− 0.77, 1.71)	0.05 (− 1.28, 1.37)	0.06 (− 1.26, 1.39)	− 0.21 (− 2.08, 1.66)
Q3	**1.37 (0.13, 2.61)**	1.22 (− 0.11, 2.54)	1.19 (− 0.13, 2.51)	0.94 (− 1.07, 2.94)
Q4	**1.58 (0.33, 2.82)**	**1.75 (0.41, 3.08)**	**1.81 (0.48, 3.14)**	**2.50 (0.43, 4.56)**
Trend-p*	0.005	0.003	0.002	0.010
Average BFD per live birth, per 3 months	0.13 (− 0.03, 0.29)	**0.18 (0.01, 0.35)**	**0.20 (0.03, 0.37)**	0.23 (− 0.03, 0.49)

^*^Trend *p* values across quartiles with quartiles coded as 1–2–3–4

Median, interquartile range (IQR) and range (min–max) of quartiles: Q1: 2 months (IQR: 0–5 months, range: 0–7 months), Q2: 12 months (IQR: 9.8–13 months, range 8–17 months), Q3: 24 months (IQR: 20–26 months, range 18–30 months), Q4: 42 months (IQR: 36–57 months, range 31–100 months)

Model 1. Unadjusted

Model 2. Adjusted for race/ethnicity, education, marital status, ever smoking, household income at enrollment, and age at 1st pregnancy

Model 3. Model 2 + age at handgrip strength measurement (precision variable)

Model 4. Model 3 + 1st trimester AHEI-P (continuous, units) and pre-pregnancy total PA (continuous, hours/week) (Restricted to women who were nulliparous at index pregnancy, N = 304, see Additional file 3: Table S2 for βs for Model 1–3 for nulliparious women only)

Bold indicates statistical significance

A participant's average lifetime BFD per live birth was calculated by taking their total lifetime BFD in months and dividing by their number of live births

We tested for potential effect modification of the association by race/ethnicity. Based on the *p* value of the interaction term between breastfeeding duration and race/ethnicity, we did not find evidence for effect modification (*p* value = 0.484 for dominant hand, *p* value = 0.447 for non-dominant hand); therefore, the final models include all women and were not stratified by race/ethnicity.

Finally, we assessed average breastfeeding duration per birth (instead of lifetime breastfeeding duration) as a standardized indicator of breastfeeding duration irrespective of parity. We found a similar positive association between lifetime breastfeeding duration and handgrip strength, suggesting that our findings are not driven by the number of children a woman has had. For instance, each 3-month increment in average breastfeeding duration per birth was associated with a 0.15 kg (95% CI − 0.03, 0.34) increase in handgrip strength for the dominant hand and 0.20 kg (95% CI 0.03, 0.37) for the nondominant hand, adjusting for race/ethnicity, marital status, ever smoking, household income at enrollment, age at first pregnancy, and age at the hand grip strength measurement (Model 3).

## Discussion

We found a positive association of lifetime breastfeeding duration with handgrip strength in midlife, with slightly stronger associations in the nondominant hand. In both unadjusted models and after adjusting for race/ethnicity, marital status, ever smoking, household income at enrollment, age at first pregnancy, and age at the handgrip strength measurement, we found that each 3-month increment in lifetime breastfeeding duration was associated with 0.10 kg higher handgrip strength for both the dominant and nondominant hand. Participants who had the highest quartile of lifetime breastfeeding duration (IQR: 36–57 months) had 1.81 kg (95% CI 0.48, 3.14) higher handgrip strength in the nondominant hand compared to those with the lowest quartile of breastfeeding duration (IQR: 0–5 months). These associations remained consistent and statistically significant with additional adjustment for diet and physical activity prior to pregnancy. Previous studies evaluating handgrip strength have identified that a decrease in handgrip strength is associated with subsequent adverse health outcomes [31, 32], suggesting longer lifetime breastfeeding duration could prevent adverse health outcomes.

To our knowledge, this study is the first to examine the relationship between total lactation duration across all pregnancies and midlife handgrip strength in US women. Handgrip strength has been established as a marker of overall health, as well as specific aspects of physiological function including muscle mass, muscle function and prognosis that decline with age. In this analysis, we found that the highest (IQR: 36–57 months) versus lowest (IQR: 0–5 months) quartile of lifetime breastfeeding duration correlated with a ~ 1 kg difference in handgrip strength. In the Prospective Urban Rural Epidemiological (PURE) study, Leong et al. found that a 5 kg decrease in handgrip strength corresponded with 10% to 20% higher odds of cardiovascular and all-cause mortality [31]. Participants in the PURE study had a median (IQR) age of 50 years (42–58) [31]. Celis-Morales et al. also found a hazard ratio of all-cause mortality of 1.16 for men and 1.20 for women with a 5 kg decrease in hand grip strength [32]. Therefore, while modest, the effect size we observed may be of relevance to chronic disease precursors that predicate clinical disease endpoint and mortality, especially since these differences are expected to increase with age. When treating breastfeeding duration as a categorical variable, we noted stronger associations of the third and fourth quartiles of lifetime breastfeeding duration with midlife handgrip strength. This finding may suggest a threshold effect wherein women reap the most benefits after 24 months of total lifetime breastfeeding duration. Additionally, when assessing average breastfeeding duration per birth as the exposure, we found similar trends of association with stronger findings in the nondominant hand, and the effect of breastfeeding duration per birth did not differ significantly by the order of pregnancy.

One proposed mechanism by which lactation may affect age-related changes in musculoskeletal function is through hormonal changes that occur during lactation. For example, estrogen, which increases during breastfeeding, may prevent muscle mass loss [3]. Messier et al. suggested that estrogen could have a direct effect on muscle mass due to the link between low estrogen levels and decreased protein synthesis [4]. Additionally, previous studies reported that increased estrogen may protect against loss of muscle mass, by mitigating inflammatory processes implicated in sarcopenia. [33–35]

### Strengths and limitations

Some strengths of our study include a larger sample size compared to previous studies and a longer follow up period which captures lifetime breastfeeding history prior to outcome assessment. Additionally, access to covariates including race/ethnicity, education, marital status, smoking, household income at enrollment, age at first pregnancy and age at handgrip strength measurement reduces potential confounding bias.

A limitation of this study is the lack of baseline data on the women’s handgrip strength prior to pregnancies. Without this information, we cannot determine the change in handgrip strength after the exposure. A source of potential bias includes unmeasured confounders such as occupation, which could be related to both ability and duration of breastfeeding, as well as handgrip strength. For example, women working in sedentary, white-collar jobs may have the resources and energy to breastfeed for longer than mothers working in more physically demanding jobs. Additionally, given that Project Viva comprises predominantly of white, college educated, married women and all had health care and insurance at the time of study enrollment, our results may be generalizable only to populations with similar demographics. Also, having smaller number of non-white participants may have reduced the power to detect effect modification by race/ethnicity.

## Conclusion

Our study suggests that there is a beneficial dose-dependent relationship between lifetime breastfeeding duration and musculoskeletal function when using handgrip strength as a measure of muscle strength. Future studies are warranted to explore how breastfeeding duration affects overall health long term.

## Supplementary Information

**Additional file 1. Figure S1: **flow chart of Project Viva women included in this analysis.

**Additional file 2. Table S1: **Characteristics of Project Viva participants included vs excluded from this analysis.

**Additional file 3.Table S2: **Associations of lifetime breastfeeding duration (BFD) with dominant and nondominant handgrip strength among N=304 women who were nulliparous at enrollment.

## Data Availability

The datasets generated and/or analyzed during the current study are not publicly available because they contain information that could compromise research participant privacy but are available from the corresponding author on reasonable request.
