# Supplementary material for: Association of total lifetime breastfeeding duration with midlife handgrip strength: findings from Project Viva
Source: BMC Womens Health. 2022 Jul 23;22:306. doi: 10.1186/s12905-022-01880-1 (PMC9308919; doi:10.1186/s12905-022-01880-1)
Supplement: Supplementary file 1 — Additional file 1. Figure S1: flow chart of Project Viva women included in this analysis. [file 12905_2022_1880_MOESM1_ESM.docx]

**Figure S1.** Flow chart of Project Viva women included in this analysis

1,257 do not have any midlife data

2100 women enrolled

167 do not have handgrip strength data, performed at in-person visits

843 women have any midlife data

676 women with handgrip strength measurement at midlife visit

45 did not provide lifetime breastfeeding duration

631 provided lifetime breastfeeding duration at midlife visit

Midlife visit takes place at approximately 17-18 years from index pregnancy.

Of the 2,100 women enrolled in Project Viva, 1257 do not have any midlife data. Of 843 with midlife data, 676 attended an in-person midlife where hand grip strength was measured in kilograms using a Jamar dynamometer. Of the 676 women that provided hand grip strength measurement at the midlife visit, 45 did not provide lifetime breastfeeding duration (BFD) data. Therefore, we were left with 631 women that provided both exposure and outcome data.
