# Supplementary material for: Association of total lifetime breastfeeding duration with midlife handgrip strength: findings from Project Viva
Source: BMC Womens Health. 2022 Jul 23;22:306. doi: 10.1186/s12905-022-01880-1 (PMC9308919; doi:10.1186/s12905-022-01880-1)
Supplement: Supplementary file 2 — Additional file 2. Table S1: Characteristics of Project Viva participants included vs excluded from this analysis. [file 12905_2022_1880_MOESM2_ESM.docx]

**Table S1.** Characteristics of Project Viva participants included vs. excluded from this analysis

| **Characteristic** | **Overall** | **Excluded** | **Included** | **P-value** |
| --- | --- | --- | --- | --- |
|  | n=2100 | n=1469 | n=631 |  |
|  | N (%) | | |  |
| **Race/Ethnicity** |  |  |  | 0.237 |
| Black | 346 (17) | 256 (18) | 90 (14) |  |
| Hispanic | 152 (7) | 108 (7) | 44 (7) |  |
| Asian | 118 (6) | 79 (5) | 39 (6) |  |
| White | 1379 (66) | 953 (66) | 426 (68) |  |
| Other | 81 (4) | 51 (4) | 30 (5) |  |
| **College graduate** |  |  |  | <.0001 |
| No | 736 (35) | 581 (40) | 155 (25) |  |
| Yes | 1340 (65) | 866 (60) | 474 (75) |  |
| **Household income > $70,000/year** | | |  | 0.003 |
| No | 725 (39) | 528 (41) | 197 (34) |  |
| Yes | 1124 (61) | 745 (59) | 379 (66) |  |
| **Nulliparous at index pregnancy** | |  |  | 0.880 |
| No | 1083 (52) | 756 (51) | 327 (52) |  |
| Yes | 1017 (48) | 713 (49) | 304 (48) |  |
| **Married or Cohabiting** |  |  |  | 0.199 |
| No | 180 (9) | 133 (9) | 47 (7) |  |
| Yes | 1895 (91) | 1313 (91) | 582 (93) |  |
