# Supplementary material for: Association of total lifetime breastfeeding duration with midlife handgrip strength: findings from Project Viva
Source: BMC Womens Health. 2022 Jul 23;22:306. doi: 10.1186/s12905-022-01880-1 (PMC9308919; doi:10.1186/s12905-022-01880-1)
Supplement: Supplementary file 3 — Additional file 3.Table S2: Associations of lifetime breastfeeding duration (BFD) with dominant and nondominant handgrip strength among N=304 women who were nulliparous at enrollment. [file 12905_2022_1880_MOESM3_ESM.docx]

**Table S2.** Associations of lifetime breastfeeding duration (BFD) with dominant and nondominant handgrip strength among N=304 women who were nulliparous at enrollment

|  | Model 1 | Model 2 | Model 3 | Model 4 |
| --- | --- | --- | --- | --- |
|  | β (95% CI) | | |  |
| **Dominant hand** |  |  |  |  |
| Exposure |  |  |  |  |
| *Lifetime BFD, per 3 months* | **0.15 (0.04, 0.25)** | **0.15 (0.04, 0.27)** | **0.14 (0.03, 0.26)** | **0.15 (0.03, 0.28)** |
| *Lifetime BFD, Quartiles* |  |  |  |  |
| Q1 | 0.0 (ref) | 0.0 (ref) | 0.0 (ref) | 0.0 (ref) |
| Q2 | 0.49 (-1.31, 2.30) | 0.20 (-1.70, 2.10) | 0.04 (-1.87, 1.94) | 0.17 (-1.86, 2.19) |
| Q3 | 1.70 (-0.10, 3.49) | 1.74 (-0.20, 3.68) | 1.45 (-0.51, 3.42) | 1.18 (-0.99, 3.35) |
| Q4 | **2.69 (0.77, 4.62)** | **2.68 (0.63, 4.73)** | **2.45 (0.39, 4.52)** | **2.33 (0.10, 4.56)** |
| Trend-p* | 0.003 | 0.004 | 0.009 | 0.027 |
| Average BFD per live birth, per 3 months | 0.15 (-0.07, 0.38) | 0.15 (-0.09, 0.40) | 0.17 (-0.07, 0.42) | 0.16 (-0.12, 0.43) |
| **Nondominant hand** |  |  |  |  |
| Exposure |  |  |  |  |
| *Lifetime BFD, per 3 months* | **0.16 (0.06, 0.26)** | **0.17 (0.06, 0.28)** | **0.16 (0.05, 0.26)** | **0.15 (0.03, 0.26)** |
| *Lifetime BFD, Quartiles* |  |  |  |  |
| Q1 | 0.0 (ref) | 0.0 (ref) | 0.0 (ref) | 0.0 (ref) |
| Q2 | 0.54 (-1.14, 2.23) | 0.08 (-1.68, 1.84) | -0.11 (-1.87, 1.64) | -0.21 (-2.08, 1.66) |
| Q3 | 1.54 (-0.14, 3.22) | 1.54 (-0.26, 3.34) | 1.20 (-0.61, 3.02) | 0.94 (-1.07, 2.94) |
| Q4 | **3.11 (1.31, 4.91)** | **3.07 (1.17, 4.97)** | **2.81 (0.91, 4.71)** | **2.50 (0.43, 4.56)** |
| Trend-p* | 0.001 | 0.001 | 0.002 | 0.010 |
| Average BFD per live birth, per 3 months | **0.23 (0.02, 0.44)** | **0.25 (0.02, 0.47)** | **0.27 (0.04, 0.49)** | 0.23 (-0.03, 0.49) |

*Trend p-values across quartiles with quartiles coded as 1-2-3-4.

Median (interquartile range): Q1 2.0 (0.0-5.0), Q2 12.0 (9.8-13.0), Q3 24.0 (20.0-26.0) and Q4 42.0 (36.0-57.0) months.

Bold indicates statistical significance.

A participant's average lifetime BFD per live birth was calculated by taking their total lifetime BFD in months and dividing by their number of live births.

Model 1. Unadjusted

Model 2. Adjusted for race/ethnicity, education, marital status, ever smoker, household income at enrollment, and age at 1st pregnancy

Model 3. Model 2 + age at handgrip strength measurement

Model 4. Model 3 + 1st trim AHEI-P (continuous, units) and pre-pregnancy total PA (continuous, hours/week)
